# Thrombose veineuse profonde étendue du membre inferieur compliquant une procréation médicalement assistée: une observation

**DOI:** 10.11604/pamj.2015.22.125.7987

**Published:** 2015-10-12

**Authors:** Amine Abdellah, Fatihi Jamal

**Affiliations:** 1 Cardiologie, 5ème Hôpital Militaire Guelmim, Maroc; 2 Médecine Interne, 5ème Hôpital Militaire Guelmim, Maroc

**Keywords:** Procréation médicalement assistée, thrombose veineuse, syndrome d′hyperstimulation ovarienne, Medically assisted procreation, venous thrombosis, Ovarian hyperstimulation syndrome

## Abstract

La survenue d'une maladie thromboembolique veineuse (MTEV) fait partie des effets indésirables de la procréation médicalement assistée (PMA). Le risque de survenue semble faible mais peu d’études ont estimé l'incidence de la MTEV en absence de syndrome d'hyperstimulation ovarienne (SHO). L'estimation du risque d’événement thromboembolique veineux chez les femmes à risque doit être identifiée avant une PMA. Nous rapportons un cas clinique d'une thrombose veineuse profonde du membre inferieur suite à une PMA sans syndrome d'hyperstimulation ovarienne.

## Introduction

Le nombre de naissance issue d'une procréation médicalement assistée (PMA) a augmenté de manière exponentielle au cours des dernières décennies. Initialement proposée pour remédier à l'infertilité des couples, les indications de la PMA ont été élargies et les techniques mieux standardisées. Parmi les complications inhérentes aux techniques de PMA le risque thrombotique est bien connu. La survenu d’événements thromboemboliques, principalement imputée à la présence d'un syndrome d'hyperstimulation ovarienne (SHO), peut se voir en dehors de ce SHO symptomatique. Nous rapportons une observation de thrombose veineuse profonde du membre inferieur au cours d'une fécondation in vitro en l'absence de SHO.

## Patient et observation

Une femme de 34 ans de race blanche ayant comme antécédents deux grossesses spontanées menées à terme en 2002 et en 2005. La patiente bénéficie d'une fécondation in vitro (FIV) en mai 2014. Le blocage ovarien débutait en phase lutéale, au 22 ^ème^ jour du cycle, par des injections quotidiennes, sous-cutanées, de triptoréline (Decapeptyl) à la dose de 0,1 mg. Quinze jours plus tard, le blocage était confirmé à l’échographie endovaginale, la stimulation ovarienne débutait par des injections quotidiennes, sous-cutanées, de follitropine alpha. A ce moment, les injections de triptoréline étaient diminuées de moitié (0,05 mg/j), jusqu'au déclenchement de l'ovulation. La stimulation ovarienne par FSH recombinante (Follitropine alpha) débutait à la dose de 300 UI/j. le déclenchement de l'ovulation avait lieu en présence de trois follicules de 19 mm de diamètre, par une injection intramusculaire de gonadotrophine chorionique ou hCG (Gonadotrophine Chorionique Endo) à la dose de 10 000 UI.. La ponction ovocytaire réalisée 36 heures après l'injection d'hCG, le transfert embryonnaire deux jours après la ponction ovocytaire. La patiente est mise au repos associé à une anticoagulation préventive durant 05 jours. Le dosage des β HCG élevés et l'examen par une échographie pelvienne après 1 mois a confirmé l’évolution d'une grossesse gémellaire. A neuf semaines d'aménorrhée, le tableau se complique d'un œdème du membre inferieur gauche. A l'examen, on retrouve une patiente en bon état général. Elle pèse 80 kg avec une taille de 1m62 soit un indice de masse corporel à 30 kg/m^2^. Un œdème douloureux du pied remontant jusqu’à la hanche affectait effectivement le membre inférieur gauche. La jambe était œdémateuse et érythémateuse. On note une perte du ballonnement du mollet et le test de Homans est positif. Le Doppler veineuse confirme une thrombose veineuse étendue de la veine surale, veine fémorale superficielle jusqu’à la veine iliaque externe du membre inferieur gauche. Le réseau artériel est perméable et de calibre normal. Echo abdomino-pelvienne montre une grossesse gémellaire évolutive. Sur le plan biologique, le bilan initial de routine est revenu normal. Le bilan de thrombophilie et du syndrome des antiphospholipides est négatif. La patiente est mise sous traitement anticoagulants héparine à bas poids moléculaire (énoxaparine 1mg/Kg à raison de deux injections par jour) durant toute la grossesse et l’évolution est progressivement favorable. La grossesse est menée à terme jusqu’à 38 semaines d'aménorrhées avec bonne évolution clinique et biologique.

## Discussion

Cette observation relate la survenue d'une thrombophlébite du membre inferieur gauche chez une patiente ayant fait l'objet d'une PMA sans SHO. La physiopathologie des manifestations thrombotiques rencontrées lors d'une stimulation hormonale n'est pas complètement élucidée à ce jour. La survenue de thromboses au cours d'une PMA est parfois la conséquence de facteurs de risque cliniques ou biologiques existant chez la patiente avant la PMA (antécédents personnels ou familiaux de thrombose, thrombophilies héréditaires, syndrome des antiphospholipides), ce qui souligne l'importance de l'identification de ces femmes à risque avant la PMA. Des modifications biologiques associées à la PMA, au SHO, aux traitements par les œstrogènes ou à la grossesse participent aussi à une augmentation du risque. Les études relatives à l'hémostase en cours de PMA sont pour la plupart anciennes, avec des méthodologies peu détaillées et la définition de SHO sévère est parfois peu précise et variable selon les auteurs. Les modifications biologiques sont similaires à celles observées pendant la grossesse ou au cours des traitements par des œstrogènes et restent souvent modérées. Elles induisent une hypercoagulabilité qui est plutôt en faveur d'une prédisposition aux thromboses veineuses. Elles sont plus prononcées en cas de SHO sévère, sont globalement corrélées à l’œstradiolémie et persistent pendant 3 semaines à 6 semaines après le début du syndrome. Peu d’études ont été conduites pour estimer le risque thromboembolique veineux dans cette situation. Le risque absolu semble faible, et l'estimation du risque thromboembolique nécessite l'inclusion de plusieurs milliers de patientes dans des cohortes de puissance suffisante. Une cohorte danoise publiée en 2012 réalisée à partir d'un registre sur 30 884 femmes entre 1994 et 2005 n'a pas mis en évidence d'augmentation statistiquement significative du risque de thromboses artérielles ou veineuses pendant les 6 mois à 12 mois suivant une PMA [1]. Aucune donnée à ce jour ne permet de déterminer si le risque est différent en fonction des protocoles de PMA. Aucune donnée ne permet d'estimer ce risque après échec de la PMA, c′est-à-dire en l'absence de grossesse.

## Conclusion

L'apparition d'une maladie thromboembolique veineuse fait partie des effets indésirables de la procréation médicalement assistée. Le risque de survenue semble faible mais peu d’études ont estimé l'incidence de la maladie thromboembolique veineuse en cas de PMA en dehors d'une hyperstimulation ovarienne d'où la nécessité de l'identification de ces femmes à risque avant une procréation médicalement assistée.
